# The Emerging Role of Non-coding RNAs in Drug Resistance of Ovarian Cancer

**DOI:** 10.3389/fgene.2021.693259

**Published:** 2021-08-20

**Authors:** Hua Lan, Jing Yuan, Da Zeng, Chu Liu, Xiaohui Guo, Jiahui Yong, Xiangyang Zeng, Songshu Xiao

**Affiliations:** Department of Obstetrics and Gynecology, Third Xiangya Hospital of Central South University, Changsha, China

**Keywords:** ovarian cancer, drug resistance, microRNA, long non-coding RNA, circular RNA

## Abstract

Ovarian cancer is one of the most common gynecological malignancies with highest mortality rate among all gynecological malignant tumors. Advanced ovarian cancer patients can obtain a survival benefit from chemotherapy, including platinum drugs and paclitaxel. In more recent years, the administration of poly-ADP ribose polymerase inhibitor to patients with BRCA mutations has significantly improved the progression-free survival of ovarian cancer patients. Nevertheless, primary drug resistance or the acquisition of drug resistance eventually leads to treatment failure and poor outcomes for ovarian cancer patients. The mechanism underlying drug resistance in ovarian cancer is complex and has not been fully elucidated. Interestingly, different non-coding RNAs (ncRNAs), such as circular RNAs, long non-coding RNAs and microRNAs, play a critical role in the development of ovarian cancer. Accumulating evidence has indicated that ncRNAs have important regulatory roles in ovarian cancer resistance to chemotherapy reagents and targeted therapy drugs. In this review, we systematically highlight the emerging roles and the regulatory mechanisms by which ncRNAs affect ovarian cancer chemoresistance. Additionally, we suggest that ncRNAs can be considered as potential diagnostic and prognostic biomarkers as well as novel therapeutic targets for ovarian cancer.

## Background

Ovarian cancer is one of the most deadly gynecologic malignancy, there are approximately 313,959 new cases and more than 207,252 deaths annually worldwide (Sung et al., 2021). Unfortunately, due to lack of effective early screening methods, 5-year survival rate was only 20–40% (Lheureux et al., 2019). Currently, the main methods used for clinical treatment of ovarian cancer are still based on cytoreductive surgery and multidrug combination chemotherapy based on platinum drugs (Armstrong et al., 2021). Chemotherapy is the main treatment option available for advanced or recurrent ovarian cancers, and the commonly used chemotherapeutic agents include platinum drugs and paclitaxel (PTX). In addition, the administration of poly-ADP ribose polymerase inhibitor (PARPi) to BRCA mutation patients has significantly improved the progression-free survival (PFS) of ovarian cancer (Tew et al., 2020). Although chemotherapy in combination with targeted therapy prolongs the overall survival of ovarian cancer patients, acquired multidrug resistance (MDR) hinders its clinical benefits. Therefore, patients with ovarian cancer frequently have a poor prognosis. The complicated mechanisms involved in MDR ovarian cancer include decreased drug uptake into the cell, increased drug efflux, intracellular drug inactivation, DNA damage repair, resistance to drug-induced apoptosis, activation of cancer stem cells, and epithelial-mesenchymal transition (EMT) (Christie et al., 2019; Liang et al., 2019; Belur Nagaraj et al., 2021; Chiappa et al., 2021). While progress has been made in understanding the pathogenesis of ovarian cancer, the detailed mechanisms of MDR remain elusive.

Non-coding RNAs (ncRNAs) are a kind of DNA transcription product that cannot be encoded into proteins. NcRNAs can be classified according to their length and shape into tiny/short ncRNAs, long ncRNAs (lncRNAs) which is larger than 200 nucleotides (nt), and circular RNA (circRNAs). Various small ncRNAs have been identified, such as microRNAs (miRNAs), PIWI-interacting RNAs (piRNAs), small nucleolar RNAs (snoRNAs), and small nuclear RNAs (snRNAs) (Kristensen et al., 2019; Shuai et al., 2019; Jin et al., 2020; Cui et al., 2021; Han et al., 2021; Luo et al., 2021; Tsitsipatis et al., 2021). NcRNAs have been proven to have important regulatory potential, both in transcription and post transcription, instead of just being “transcription noise” or “transcription garbage.” There is ample evidence that ncRNAs are of crucial importance in the regulation of gene expression. Meanwhile, ncRNAs participate in many biological functions, such as cell proliferation, cell cycle progression, and apoptosis (Cocquerelle et al., 1993; Memczak et al., 2013; Cech and Steitz, 2014; Li et al., 2021; Ramat and Simonelig, 2021; Statello et al., 2021). In addition, a large number of studies have shown that abnormally expressed ncRNAs participate in tumor cell invasion, metastasis, drug resistance and radiotherapy resistance (Bi et al., 2020; Chen et al., 2020; Wang P. et al., 2020). Similarly, previous research suggested that ncRNAs are dysregulated when drug resistance develops, which indicates that in ovarian cancer, multiple ncRNAs might play a vital role in drug resistance.

**FIGURE 1:**
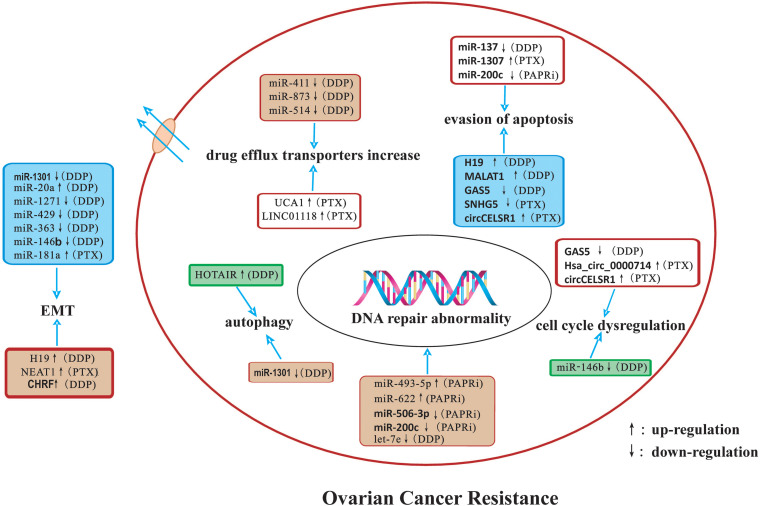
A summary diagram of miRNAs, lncRNAs, and circRNAs involved in the drug resistance of ovarian cancer. Several ncRNAs could participate in drug resistance of ovarian cancer by influencing cell apoptosis, proliferation, cell cycle, autophagy, DNA repair, and epithelial-mesenchymal transition through modulating the expression of downstream target genes and related signaling pathway.

In this review, we summarized the detailed mechanisms by which miRNAs, lncRNAs, and circRNAs affect ovarian cancer drug resistance. The potential mechanisms of ncRNAs related to drug-resistance in ovarian cancer are summarized in Figure 1. NcRNAs have potential as diagnostic and prognostic biomarkers as well as novel therapeutic targets for ovarian cancer in the future.

## MiRNAs and Drug Resistance

MicroRNAs are a class of small ncRNAs containing 20–24 nt that can post transcriptionally suppress gene expression by binding to the 3′-untranslated region (3′-UTR) of multiple target messenger RNAs (mRNAs) and/or other RNAs (Wang X. et al., 2021). MiRNAs are key molecules that are involved in many different kinds of fundamental cellular processes, including cell differentiation and proliferation, cell cycle regulation, angiogenesis, metabolic stress, and other functions (He et al., 2019; Komoll et al., 2021; Xing et al., 2021). It has been found that multiple miRNAs are dysregulated in ovarian cancer and are closely related to its occurrence, development, metastasis and drug resistance (Mak et al., 2017; Tung et al., 2020; Zhang Z. et al., 2020). Significant changes in miRNA expression profiles have been observed in drug-resistant cancer cells in comparison with parental drug-sensitive cancer cells. The involvement of miRNAs in ovarian cancer resistance to platinum drugs, PTX, ADR, and PARPi is summarized below.

## MiRNAs and Resistance to Platinum

Platinum drugs are cell cycle non-specific drugs that are widely used in the clinic. They induce DNA damage or ribosome biosynthesis stress and activate tumor cell death by apoptosis or necrosis. However, a series of complex mechanisms lead to platinum resistance (Bruno et al., 2017; Huang et al., 2019). The commonly used platinum drugs include the first generation of drug cisplatin (DDP), the second generation of drug carboplatin, as well as the third-generation drugs oxaliplatin and Lopatin. Many miRNAs are related to the resistance to platinum drugs in ovarian cancer (Table 1).

**TABLE 1:** MiRNAs and platinum resistance in ovarian cancer.

**MiRNAs**	**Expression^1^**	**Genes and pathways**	**Drugs**	**References**
miR-205-5p	↑	PTEN/Akt	Cisplatin	Jin et al., 2018
miR-216a	↑	PTEN	Cisplatin	Shi et al., 2018
miR-483-3p	↑	PKC-alpha	Cisplatin	Arrighetti et al., 2016
miR-224-5p	↑	PRKCD	Cisplatin	Zhao et al., 2014
miR-30b	↑	MYPT1	Cisplatin	Munoz-Galvan et al., 2020
miR-149-5p	↑	MST1, SAV1	Cisplatin	Xu M. et al., 2018
miR-1180	↑	SFRP1	Cisplatin	Gu et al., 2019
miR-493-5p	↑	BRCA2	Cisplatin	Meghani et al., 2018
miR-31	↑	KCNMA1	Cisplatin	Samuel et al., 2016
miR-98-5p	↑	CDKN1A, Dicer1	Cisplatin	Wang Y. et al., 2018; Guo et al., 2019
miR-551b	↑	FOXO3, TRIM31	Cisplatin	Wei et al., 2016
miR-20a	↑	Vimentin, E-cadherin, N-cadherin	Cisplatin	Liu et al., 2017
miR-411	↓	ABCG2	Cisplatin	Chen et al., 2018
miR-873	↓	ABCB1	Cisplatin	Wu et al., 2016
miR-514	↓	ABCA1, ABCA10, ABCF2	Cisplatin	Xiao et al., 2018
miR-1301	↓	E-cadherin, N-cadherin, ATG5, Beclin1	Cisplatin	Yu and Gao, 2020
miR-1271	↓	E-cadherin, N-cadherin, α-SMA	Cisplatin	Chen Y. et al., 2019
miR-429	↓	ZEB1	Cisplatin	Zou et al., 2017
miR-363	↓	Snai1	Cisplatin	Cao et al., 2018
miR-146b	↓	Vimentin, ZEB1, cyclin D1	Cisplatin	Yan et al., 2018
miR-142-5p	↓	XIAP, BIRC3, BCL2, BCL2L2, MCL1	Cisplatin	Li X. et al., 2019
miR-335-5p	↓	BCL2L2	Cisplatin	Li et al., 2017a
miR-146a-5p	↓	XIAP, BCL2L2, BIRC5	Cisplatin	Li et al., 2017b
miR-137	↓	XIAP, EZH2	Cisplatin	Liu R. et al., 2018; Sun et al., 2019
miR-708	↓	IGF2BP1/Akt	Cisplatin	Qin et al., 2017
miR-503	↓	PI3K/Akt	Cisplatin	Wu et al., 2018
miR-199	↓	PTPN3	Cisplatin	Li S. et al., 2016
miR-199a-3p	↓	ITGB8, DDR1	Cisplatin	Deng et al., 2017; Cui et al., 2018
let-7d-5p	↓	HMGA1	Cisplatin	Chen Y. N. et al., 2019
let-7e	↓	BRCA1, Rad51	Cisplatin	Xiao et al., 2017
miR-200b/c	↓	DNMT3A/DNMT3B/DNMT1	Cisplatin	Liu et al., 2019
miR-515-3p	↓	AXL	Oxaliplatin	Hisamatsu et al., 2019
let-7d-3p	↑	ABC transporters, HIF-1, RAS, ErbB	Carboplatin	Garcia-Vazquez et al., 2018
miR-34c-5p	↓	AREG-EGFR-ERK	Carboplatin	Tung et al., 2017

*^1^MiRNAs either up-regulated (↑) or down-regulated (↓) in platinum resistant ovarian cancer cells. This table shows 34 miRNAs whose expression levels and potential targets in platinum resistance of ovarian cancer.*

Several oncogenic miRNAs can promote resistance to platinum drugs in ovarian cancer cells. For example, miR-205-5p and miR-216a confer DDP resistance by suppressing the PTEN (phosphatase and tensin homolog)/Akt signaling pathway in ovarian cancer cells (Jin et al., 2018; Shi et al., 2018). Similarly, miR-483-3p and miR-224-5p have also been found to promote DDP resistance by silencing protein kinase C (PRKC) family members (Zhao et al., 2014; Arrighetti et al., 2016). Studies have shown that miR-30b and miR-149-5p are involved in the Hippo signaling pathway and promote DDP resistance by downregulating the target genes protein phosphatase 1 regulatory subunit 12A (PPP1R12A), STE20-like kinase 1 (MST1), and protein salvador homolog 1 (SAV1), respectively (Xu M. et al., 2018; Munoz-Galvan et al., 2020). In addition, oncogenic miR-1180, miR-493-5p, and miR-31 confer DDP resistance to ovarian cancer cells through silencing secreted frizzled-related protein 1(SFRP1), BRCA2, and potassium calcium-activated channel subfamily M alpha 1 (KCNMA1), respectively (Samuel et al., 2016; Meghani et al., 2018; Gu et al., 2019). In animal models, miR-98-5p can potentiate the resistance of ovarian cancer to DDP, suggesting that miR-98-5p is a possible therapeutic target of ovarian cancer (Wang Y. et al., 2018; Guo et al., 2019). MiR-551b functions through the suppression of forkhead box O3 (FOXO3) and tripartite motif containing 31 (TRIM31), two important tumor suppressors. It was also found that elevated expression of miR-551b is significantly associated with worse survival of xenograft ovarian cancer models (Wei et al., 2016). Additionally, miR-20a could enhance DDP resistance of OVCAR3 ovarian cancer cells by altering the expression of EMT markers (E-cadherin, N-cadherin, and vimentin) (Liu et al., 2017).

In contrast, multiple tumor suppressor miRNAs have been found to be able to reverse DDP resistance in ovarian cancer. For instance, tumor suppressors miR-411, miR-873, and miR-514 have been confirmed to be involved in DDP resistance of ovarian cancer by modulating the expression/function of the ABC transporters family members (Wu et al., 2016; Chen et al., 2018; Xiao et al., 2018). In the meantime, miR-1301, miR-1271, miR-429, miR-363, and miR-146b can sensitize ovarian cancer cells to DDP by inhibiting the expression of multiple EMT-related genes (Zou et al., 2017; Cao et al., 2018; Yan et al., 2018; Chen Y. et al., 2019; Yu and Gao, 2020). By inhibiting the Bcl-2 signaling pathway, several tumor suppressor miRNAs, including miR-142-5p, miR-335-5p, miR-146a-5p, and miR-137 have been confirmed to sensitize ovarian cancer cells to DDP (Li et al., 2017a,b; Liu R. et al., 2018; Li X. et al., 2019). In addition, exogenous expression of miR-137 can also strongly promote DDP chemosensitivity through downregulating the expression of X-linked inhibitor of apoptosis (XIAP) and the zeste homolog 2 (EZH2) (Sun et al., 2019). Similarly, miR-708 and miR-503 can modulate ovarian cancer resistance to cisplatin through regulating the Akt pathway (Qin et al., 2017; Wu et al., 2018).Recently, emerging evidence has shown that miRNAs are aberrantly expressed in ovarian cancer, and some of them regulate different mRNAs and inhibit cisplatin resistance. Abnormal expression of the miR-199 cluster, for example, has been confirmed to increase the sensitivity of ovarian cancer cells to DDP through silencing the expression of protein tyrosine phosphatase non-receptor type 3 (PTPN3), integrin subunit beta 8 (ITGB8) and discoidin domain receptor 1 (DDR1) (Li S. et al., 2016; Deng et al., 2017; Cui et al., 2018). Additionally, ectopic miR-let-7 cluster expression can weaken DDP resistance in ovarian cancer cells by inhibiting high mobility group AT-hook 1 (HMGA1), RAD51 recombinase (RAD51), and BRCA1, indicating that the miR-let-7 cluster might be a candidate biomarker to predict ovarian cancer responders to DDP treatment (Xiao et al., 2017; Chen Y. N. et al., 2019). Moreover, the miR-200b/c cluster can improve the sensitivity of ovarian cancer cells to cisplatin by inhibiting the expression of DNA methyltransferase (DNMT) (Liu et al., 2019).

Studies on carboplatin and oxaliplatin are far less extensive than cisplatin. Tumor suppressor miR-515-3p can regulate oxaliplatin sensitivity by targeting AXL Receptor Tyrosine Kinase (AXL) (Hisamatsu et al., 2019). Similarly, let-7d-3p could enhance carboplatin-resistance (Garcia-Vazquez et al., 2018). Tumor suppressors miR-634 and miR-34c-5p have been proven to be involved in the regulation of carboplatin sensitivity through the MAPK pathway (Tung et al., 2017).

## MiRNAs and PTX Resistance

Paclitaxel is one of the first-line chemotherapy drugs used to treat ovarian cancer. It is highly cytotoxic against tubulin. It induces and promotes the polymerization of tubulin and microtubule assembly, and it prevents depolymerization, stabilizing microtubules, and inhibiting the mitosis of cancer cells, leading to cell cycle arrest in G2/M. This effectively prevents the proliferation of cancer cells. It has been reported that various miRNAs are involved in PTX-resistance of ovarian cancer (Table 2).

**TABLE 2:** MiRNAs and paclitaxel resistance in ovarian cancer.

**MiRNAs**	**Expression^1^**	**Genes and pathways**	**References**
miR-21	↑	APAF1	Au Yeung et al., 2016
miR-630	↑	APAF1	Eoh et al., 2018
miR-1307	↑	CIC, ING5	Chen W. T. et al., 2017; Zhou et al., 2019
miR-181a	↑	E-cadherin, N-cadherin	Li L. et al., 2016
miR-215	↓	XIAP	Ge et al., 2016
miR-200bc/141	↓	EMT	Duran et al., 2017
miR-92	↓	DKK1	Chen M. W. et al., 2017
miR-503-5p	↓	CD97	Park and Kim, 2019
miR-136	↓	NOTCH3	Jeong et al., 2017
miR-383-5p	↓	TRIM27	Jiang et al., 2019
miR-874	↓	SIK2	Xia et al., 2018

*^1^MiRNAs either up-regulated (↑) or down-regulated (↓) in paclitaxel resistant ovarian cancer cells. This table shows 11 miRNAs whose expression levels and potential targets in paclitaxel resistance of ovarian cancer.*

Several oncogenic miRNAs can facilitate PTX resistance, such as miR-21 and miR-630. Exogenous expression of miR-21 and miR-630 enhanced PTX resistance of ovarian cancer cells by silencing apoptotic peptidase activating factor 1 (APAF1) (Au Yeung et al., 2016; Eoh et al., 2018). Similarly, miR-1307, a highly expressed miRNA in ovarian cancer tissues and cell lines, has been demonstrated to be positively correlated with PTX resistance. By targeting the capicua transcriptional repressor (CIC) and the inhibitor of growth family member 5 (ING5), miR-1307 could dramatically inhibit apoptosis induced by PTX (Chen W. T. et al., 2017; Zhou et al., 2019). Moreover, the miR-181a level in chemoresistant cancer tissues is significantly higher than in chemosensitive cancer tissues and in normal tissue, and its upregulation is associated with an increased level of EMT and decreased cell apoptosis induced by PTX treatment (Li L. et al., 2016).

In contrast, several tumor suppressor miRNAs may reverse PTX resistance in ovarian cancer. The Bcl-2 family participates in the chemoresistance of malignancies, including ovarian cancer. Tumor suppressors miR-215 can promote PTX-induced apoptosis of ovarian cancer cells by silencing the expression of XIAP (Ge et al., 2016). Activation of the EMT pathway has also been observed to regulate PTX resistance of ovarian cancer. A variety of miRNAs, such as miR-200b and miR-200c, have been observed to be involved in the EMT pathway mediated PTX resistance of ovarian cancer (Duran et al., 2017). By inhibiting the signal transducer and activator of transcription 3 (STAT3) signaling pathway, several tumor suppressor miRNAs, including miR-92 and miR-503-5p, have been found to sensitize ovarian cancer cells to PTX. In animal models, targeting STAT3 in combination with paclitaxel can synergistically reduce intraperitoneal dissemination and prolong the survival of mice with ovarian cancer (Chen M. W. et al., 2017; Park and Kim, 2019). Similarly, tumor suppressors miR-136, miR-383-5p, and miR-874 have been reported to conquer PTX resistance of ovarian cancer cells by silencing NOTCH3, tripartite motif containing 27 (TRIM27), and salt inducible kinase 2 (SIK2), respectively (Jeong et al., 2017; Xia et al., 2018; Jiang et al., 2019).

## MiRNAs and PARPi Resistance

Poly-ADP ribose polymerase inhibitor have emerged as exciting new chemotherapy options for women with ovarian cancer, especially for patients with BRCA1 or BRCA2 mutations or non-functional homologous recombination repair pathways. The most advantageous feature of PARPi is its mechanism of action. PARPi is able to eliminate the function of PARP, leading to the accumulation of single-stranded breaks (SSB), which in turn can be converted into double-strand breaks (DSB) that the cell cannot repair, leading to cancer cell death (Wiltshire et al., 2010). Moreover, PARPi can enhance the efficacy of radiotherapy and chemotherapy with docetaxel and platinum drugs. Three PARPis have been approved for the treatment of recurrent epithelial ovarian cancer in the United States: olaparib, rucaparib, and niraparib. However, long-term use of PARPis may cause PARPi resistance. In ovarian cancer cells, multiple miRNAs were found to be involved in PARPi resistance (Table 3).

**TABLE 3:** MiRNAs and PARPi resistance in ovarian cancer.

**MiRNAs**	**Expression^1^**	**Genes and pathways**	**References**
miR-493-5p	↑	RNASEH2A, FEN1, SSRP1	Meghani et al., 2018
miR-622	↑	Ku	Choi et al., 2016
miR-506-3p	↓	EZH2/β-catenin	Sun et al., 2021
miR-200c	↓	NRP1	Vescarelli et al., 2020

*^1^MiRNAs either up-regulated (↑) or down-regulated (↓) in paclitaxel resistant ovarian cancer cells. This table shows four miRNAs whose expression levels and potential targets in PARPi resistance of ovarian cancer.*

Multiple oncogene miRNAs can promote PARPi resistance. According to a recent report, miR-493-5p is significantly upregulated in BRCA2-mutated ovarian cancer cells and it participates in the PARPi resistance process by regulating ribonuclease H2 subunit A (RNASEH2A), flap structure-specific endonuclease 1 (FEN1), and structure specific recognition protein 1 (SSRP1). miR-493-5p can reduce single-strand annealing (SSA), stabilize the replication fork, and thus induce PARPi tolerance (Meghani et al., 2018). In addition, miR-622 is highly expressed in BRCA1-deficient high-grade serous ovarian carcinomas (HGSOCs), which can rescue the homologous recombination repair (HRR) defect of BRCA1 mutant ovarian cancer and promote PARPi resistance by regulating the expression of Ku complex and inhibiting HR and non-homologous end joining (NHEJ) (Choi et al., 2016).

In contrast, multiple tumor suppressor miRNAs can reverse the PARPi resistance of ovarian cancer. For instance, miR-506-3p acts as a vital regulator in the sensitivity to PARPis and cisplatin by targeting EZH2/β-catenin pathway in ovarian cancers (Sun et al., 2021). Additionally, ectopic miR-200c expression can increase apoptosis and weaken the resistance to olaparib in the ovarian cancer cells SKOV3/PARPi by silencing Neuropilin 1 (NRP1) (Vescarelli et al., 2020).

## LncRNAs and Therapy Resistance

Long non-coding RNAs are a category of RNA transcripts longer than 200 nt without coding capacity, which are transcribed by RNA Polymerase II (RNAP II) and expressed in a tissue-specific manner (Quinn and Chang, 2016). At present, it is known that lncRNAs can regulate the malignant biological behavior of cells by acting as a competitive endogenous RNA (ceRNA), recruiting downstream molecules, serving as protein scaffolds, transmitting regulatory signals (Wong et al., 2018), and regulating endolysosome pH (Miller et al., 2018). A number of lncRNAs have a close relationship to the development of ovarian cancer metastasis, recurrence, and chemotherapy resistance (Winham et al., 2019; Zhang M. et al., 2019; Sun et al., 2020). Aberrantly expressed lncRNAs may participate in ovarian cancer progression through various mechanisms, including inducing autophagy, increasing DNA damage repair, changing cell cycle progression and checkpoints, inducing anti-apoptosis, regulating cell signaling pathways, and promoting EMT (Liu et al., 2015; Yan et al., 2017; Xu Q. F. et al., 2018; Wu et al., 2019). Several lncRNAs have been found to be involved in drug resistance in ovarian cancer (Tables 4, 5).

**TABLE 4:** LncRNAs and platinum resistance in ovarian cancer.

**LncRNAs**	**Expression^1^**	**Genes and pathways**	**Drugs**	**References**
UCA1	↑	miR-143/FOSL2	Cisplatin	Li Z. et al., 2019
HOTAIR	↑	Wnt/β-catenin pathway	Cisplatin	Li J. et al., 2016
		NF-κB pathway	Cisplatin	Ozes et al., 2016
		ATG7	Cisplatin	Yu et al., 2018
H19	↑	EMT	Cisplatin	Liu et al., 2015
		GSH metabolism	Cisplatin	Zheng et al., 2016
		EZH2/p21/PTEN pathway	Cisplatin	Sajadpoor et al., 2018
NEAT1	↑	miR-770-5p/PARP1	Cisplatin	Zhu et al., 2020
CCAT1	↑	miR-454/survivin	Cisplatin	Wang D. Y. et al., 2020
MALAT1	↑	NOTCH1	Cisplatin	Bai et al., 2018
	↑	miR-1271-5p/E2F5	Cisplatin	Wang Y. et al., 2020
Linc00161	↑	miR-128/MAPK1	Cisplatin	Xu et al., 2019
CHRF	↑	EMT and STAT3 pathway	Cisplatin	Tan et al., 2020
ANRIL	↑	miR-324-5p/Ran axis	Cisplatin	Wang K. et al., 2021
SNHG22	↑	miR-2467/Gal-1	Cisplatin	Zhang P. F. et al., 2019
GAS5	↓	E2F4/PARP1/MAPK	Cisplatin	Long et al., 2019
PANDAR	↓	SFRS2-p53	Cisplatin	Wang H. et al., 2018
LINC01125	↓	miR-1972	Cisplatin	Guo and Pan, 2019
MEG3	↓	miR-214	Cisplatin	Zhang et al., 2017

*^1^LncRNAs either up-regulated (↑) or down-regulated (↓) in platinum resistant ovarian cancer cells. This table shows 14 lncRNAs whose expression levels and potential targets in platinum resistance of ovarian cancer.*

**TABLE 5:** LncRNAs and paclitaxel resistance in ovarian cancer.

**LncRNAs**	**Expression^1^**	**Genes and pathways**	**References**
UCA1	↑	miR-654-5p/SIK2	Li Z. Y. et al., 2020
		miR-129/ABCB1	Wang J. et al., 2018
NEAT1	↑	miR-194/ZEB1	An et al., 2017
LINC01118	↑	miR-134/ABCC1	Shi and Wang, 2018
PRLB	↑	RSF1/NF-κB	Zhao and Hong, 2021
SNHG22	↑	miR-2467/Gal-1	Zhang P. F. et al., 2019
FER1L4	↓	MAPK	Liu S. et al., 2018
SNHG5	↓	miR-23a	Lin et al., 2020

*^1^LncRNAs either up-regulated (↑) or down-regulated (↓) in paclitaxel resistant ovarian cancer cells. This table shows seven lncRNAs whose expression levels and potential targets in paclitaxel resistance of ovarian cancer.*

It has been reported that lncRNA UCA1 (urothelial cancer associated 1) is significantly upregulated in PTX-resistant ovarian cancer tissues and cell lines and confers ovarian cancer resistance to PTX. UCA1 promote tumor progression both *in vitro* and *in vivo*. SIK2 protein is involved in the separation of centrosomes during mitosis, which can lead to ovarian cancer drug resistance (Ahmed et al., 2010; Zhou et al., 2017). In ovarian cancer cells, UCA1 can induce SIK2 expression *via* endogenous sponging of miR-654-5p and thus antagonize chemosensitivity to PTX (Li Z. Y. et al., 2020). Additionally, ABCB1 (ATP binding cassette subfamily B member 1) is one of the members of the superfamily of ABC transporters that are involved in MDR. In ovarian cancer cells, UCA1 can also induce ABCB1 expression though endogenous sponging of miR-129 to enhance PTX tolerance (Wang J. et al., 2018). In recent years, lncRNA UCA1 has also been found to be involved in cisplatin resistance in ovarian cancer and blood UCA1 levels are upregulated in patients after cisplatin treatment. *Via* binding to the 3′-UTRs of FOS-like 2 (FOSL2), miR-143 can negatively regulate FOSL2 expression, suggesting that the UCA1/miR-143 axis may have potential therapeutic value for the treatment of cisplatin resistance in ovarian cancer patients (Li Z. et al., 2019).

Long non-coding RNAs HOTAIR (HOX antisense intergenic RNA) is one of the most well-studied lncRNAs, which is transcribed from the antisense strand of the HOXC gene cluster present on chromosome 12 with a length of 2.2 kb. HOTAIR, a highly expressed lncRNA in ovarian cancer tissues and cell lines, has been found to be positively correlated with advanced tumor stages, high histological grade, lymph node metastasis, drug resistance, and poor prognosis of ovarian cancer patients (Qiu et al., 2014; Wang et al., 2015). Moreover, it has been reported that exogenous HOTAIR overexpression in ovarian cancer cells significantly promoted cisplatin resistance by regulating the Wnt/β-catenin signaling pathway as well as the NF-κB-HOTAIR axis, indicating that HOTAIR may act as a regulator of cisplatin resistance (Li J. et al., 2016; Ozes et al., 2016). Similarly, knockdown of HOTAIR can inhibit autophagy *via* decreasing autophagy related 7 (ATG7) expression, and the inhibition of cisplatin-induced autophagy by silencing HOTAIR has been shown to enhance the chemotherapeutic efficacy of cisplatin in ovarian cancer (Yu et al., 2018).

Increasing findings indicate that lncRNA H19 plays an important role in chemotherapy drug resistance of ovarian cancer. In the OVCAR3/DDP resistant ovarian cancer cell, silencing lncRNA H19 can significantly increase E-cadherin expression and reduce twist, slug, and snail expression, indicating that lncRNA H19 induces cisplatin resistance *via* EMT (Wu et al., 2019). In addition, lncRNA H19 can also confer resistance to cisplatin to ovarian cancer cells by promoting glutathione (GSH) metabolism (Zheng et al., 2016). It has been reported that valproic acid (VPA) acts on A2780/CP resistant cells, which negatively regulates the expression of lncRNA H19, and then induces cell apoptosis and inhibits cell proliferation, thereby making A2780 resistant cells sensitive to cisplatin (Sajadpoor et al., 2018). These findings suggest that lncRNA H19 has potential as a new target for overcoming drug resistance in ovarian cancer.

Long non-coding RNAs NEAT1 (nuclear paraspeckle assembly transcript 1) was reported to be correlated with clinically poor paclitaxel response ovarian cancer. It has been found that lncRNA NEAT1 promotes paclitaxel resistance *via* competitively binding miR-194 to facilitate ZEB1 expression in ovarian cancer cells (An et al., 2017). Recently, LncRNA NEAT1 is also found to play a part in cisplatin resistance of ovarian cancer. NEAT1 is significantly upregulated in ovarian cancer, associates with cisplatin resistance and FIGO stage. Knockdown of NEAT1 suppresses cisplatin resistance of ovarian cancer cells *in vitro* and *in vivo*. LncRNA NEAT1 contributes to DDP resistance of ovarian cancer cells by regulating PARP1 expression *via* miR-770-5p (Zhu et al., 2020).

In addition, some other lncRNAs were found to be involved in platinum-based chemotherapy resistance in ovarian cancer. On the one hand, lncRNAs can promote platinum resistance. For instance, lncRNA CCAT1 (colon cancer associated transcript 1) is upregulated in A2780/DDP and SKOV3/DDP resistant ovarian cancer cells, and it can confer resistance to DDP by modulating the miR-454/survivin axis (Wang D. Y. et al., 2020). LncRNA metastasis-associated lung adenocarcinoma transcript 1 (MALAT1) has been reported to be upregulated and to contribute to ovarian cancer tumorigenesis. Knockdown of MALAT1 could enhance cisplatin-induced apoptosis and improve the chemosensitivity of ovarian cancer cells to cisplatin through inhibiting the notch1 signaling pathway (Bai et al., 2018). Besides, MALAT1 could regulate ovarian cancer progression and DDP- resistance by miR-1271-5p/E2F5 Axis (Wang Y. et al., 2020). Moreover, it has been found that lncRNA linc0161 functions as a ceRNA of microRNA-128 and promotes drug resistance through blocking MAPK1 (Xu et al., 2019). In addition, CHRF contributes to cisplatin resistance of ovarian cancer cells by regulating EMT and STAT3 signaling *via* miR-10b (Tan et al., 2020). ANRIL could modulate the progression, drug resistance and tumor stem cell-like characteristics of ovarian cancer cells *via* miR-324-5p/Ran Axis (Wang K. et al., 2021).

Some tumor suppressor lncRNAs can reverse platinum drug resistance of ovarian cancer. LncRNA GAS5 expression in SKOV3/DDP cells has been found to be significantly reduced compared to that in drug-sensitive cells, and it has been reported that GAS5 can sensitize ovarian cancer cells to DDP by leading to G0/G1 cell cycle arrest and increasing apoptosis. Further research showed that GAS5 could inhibit DDP-resistance and tumor progression of ovarian cancer *via* the GAS5-E2F4-PARP1-MAPK axis (Long et al., 2019). It has been reported that lncRNA PANDAR dictates the chemoresistance of ovarian cancer by regulating SFRS2-mediated p53 phosphorylation (Wang H. et al., 2018). Interestingly, lncRNA linc01125 can inhibit ovarian cancer cell proliferation and it enhances the cytotoxicity of DDP in ovarian cancer cells. Tumor suppressor linc01125 has been shown to enhance the cisplatin sensitivity of ovarian cells by sponging miR-1972 (Guo and Pan, 2019). In addition, the literature shows that curcumin inhibits cisplatin resistance development partly by regulating extracellular vesicle-mediated transfer of MEG3 and miR-214 in ovarian cancer (Zhang et al., 2017).

There are several novel lncRNAs that have been found to play crucial functions in ovarian cancer PTX resistance. For instance, it has been reported that lncRNA linc0118 is significantly upregulated in PTX-resistant ovarian cancer tissues and cell lines and confers ovarian cancer resistance to PTX. Linc0118 can promote tumor progression *in vitro* and *in vivo*. In ovarian cancer cells, linc0118 can induce ABCC1 expression *via* endogenous sponging of miR-134 and, thus, antagonize chemosensitivity to PTX (Shi and Wang, 2018). In ovarian cancer cells, lncRNA-PRLB have been found to promote TAX resistance by suppressing miR-150-5p and activating NF-κB signaling. Moreover, PRLB has been found to inhibit TAX in ovarian cancer cells through enhancing RSF1 expression, whereas elevated PRLB expression has been found to be associated with a poor response to TAX treatment (Zhao and Hong, 2021). LncRNA SNHG22 is another chemoresistance-related gene and it has been found to promote DDP resistance and PTX resistance through regulating the miR-2467/galectin 1 (Gal-1) axis and it is correlated with poor patient outcomes (Zhang P. F. et al., 2019).

In contrast, a number of tumor suppressor lncRNAs can reverse PTX drug resistance in ovarian cancer. In comparison with normal ovarian epithelial cells, lncRNA FER1L4 is downregulated in SKOV3/PTX resistant cells. Overexpression of the lncRNA FER1L4 can inhibit paclitaxel tolerance of ovarian cancer cells through regulating MAPK signaling pathway (Liu S. et al., 2018). Recently, significantly diminished expression of lncRNA SNHG5 was observed in SKOV3/PTX and HeyA-8/PTX PTX-resistant ovarian cancer cells. Exogenous expression of lncRNA SNHG5 has been found to promote apoptosis, inhibit cell proliferation and enhance PTX sensitivity of ovarian cancer cells by sponging miR-23a (Lin et al., 2020).

## CircRNAs and Chemoresistance in Ovarian Cancer

Circular RNAs are crucial members of the ncRNA family, and those related to animal physiologies have been widely studied in recent years. CircRNAs have a closed-loop structure because of a covalent junction between their 3′ and 5′ ends. CircRNAs show stability, conservation, abundance, and tissue and cell specificity (Salzman et al., 2013; Ashwal-Fluss et al., 2014; Maass et al., 2017; Xia et al., 2017). CircRNAs play important roles in biological functions by acting as a “microRNA sponge,” regulating gene transcription and interacting with RNA binding proteins in most cases (Fan et al., 2021; Shen et al., 2021; Zeng et al., 2021). Accumulating evidences have shown that circRNAs are abnormally expressed in various malignant tumors, and circRNAs can act as both proto-oncogenes and tumor suppressors. It has been reported that circRNAs in tumors not only contribute to multiple processes of malignancy, including cell differentiation, proliferation, invasion, and metastasis but are also involved in the mechanism of chemotherapy resistance (Ding et al., 2020; Hong et al., 2020; Ou et al., 2020; Table 6).

**TABLE 6:** CircRNAs and drug resistance in ovarian cancer.

**CircRNAs**	**Expression^1^**	**Genes and pathways**	**Drugs**	**References**
circTNPO3	**↑**	miR-1299/NEK2	Paclitaxel	Xia et al., 2020
circNRIP1	↑	miR-211-5p/HOXC8	Paclitaxel	Li M. et al., 2020
Hsa_circ_0000714	↑	miR-370-3p/RAB17	Paclitaxel	Guo et al., 2020
CELSR1	↑	miR-1252/FOXR2	Paclitaxel	Zhang S. et al., 2020
circEXOC6B	**↓**	miR-376c-3p/FOXO3	Paclitaxel	Zheng et al., 2020
Cdr1as	**↓**	miR-1270/SCAI	Cisplatin	Zhao et al., 2019
circFoxp1	**↑**	miR-22/CEBPG, miR-150-3p/FMNL3	Cisplatin	Luo and Gui, 2020

*^1^CircRNAs either up-regulated (↑) or down-regulated (↓) in chemo-resistant ovarian cancer cells. This table shows seven circRNAs whose expression levels and underlying pathways in chemoresistance of ovarian cancer.*

Several circRNAs are known to be involved in PTX-resistant ovarian cancer. The cancer-related circTNPO3 has, for example, been found to function as an oncogene in ovarian cancer and confer PTX resistance. CircTNPO3 associates with advanced FIGO stage and histological type. CircTNPO3 promotes PTX resistance of ovarian cancer cells *in vitro* and *in vivo*. CircTNPO3 promotes PTX resistance *via* competitively binding miR-1299 to upregulate NEK2 (Xia et al., 2020). Moreover, circNRIP1 was up-regulated in PTX-resistant ovarian cancer tissues and cells. Silencing of circNRIP1 suppressed the PTX resistance of ovarian cancer cells *in vitro* and *in vivo*. Oncogenic CircNRIP1 could contribute to PTX resistance of ovarian cancer by modulating expression of the miR-211-5p/HOXC8 axis (Li M. et al., 2020). Additionally, Hsa_circ_0000714 is an up-regulated circRNA in PTX resistant cells SKOV3/PTX and A2780/PTX, which is contributed to PTX resistance by influencing cell cycle G1/S transition and colony formation. Hsa_circ_0000714 mediates PTX resistance in ovarian cancer cells by sponging miR-370-3p and regulating the expression of RAB17 (Guo et al., 2020). Meanwhile, the cancer-related circCELSR1 (hsa_circ_0063809) has also been identified to be upregulated in SKOV3/PTX and HeyA-8/PTX PTX-resistant ovarian cancer cell lines. Inhibiting circCELSR1 can cause ovarian cancer cell cycle G0/G1 arrest and an increase in apoptosis. CircCELSR1 has been shown to contribute to PTX resistance by modulating forkhead box R2 (FOXR2) expression through miR-1252 (Zhang S. et al., 2020). On the contrary, tumor suppressor circRNAs can reverse PTX resistance in ovarian cancer. circEXOC6B shows notably decreased expression in ovarian cancer tissues and is associated with long survival time of ovarian cancer patients. In ovarian cancer cells, circEXOC6B could suppress FOXO3 expression *via* endogenous sponging miR-376c-3p and, thus, elevate chemosensitivity to PTX (Zheng et al., 2020).

Also, several circRNAs have been found to be involved in ovarian cancer DDP chemoresistance. Significantly decreased expression levels of circRNA Cdr1as have been observed in both tissues and serum exosomes of Cisplatin-Resistant ovarian cancer patients. It has been confirmed that downregulating suppressor of cancer cell invasion (SCAI) by sponging miR-1270, Cdr1as can conquer DDP resistance of ovarian cancer cells (Zhao et al., 2019). Recently, circulating exosomal circFoxp1, whose expression is positively associated with International Federation of Gynecology and Obstetrics stage, primary tumor size, lymphatic metastasis, distant metastasis, residual tumor diameter, and clinical response, has been reported to promote resistance to DDP of ovarian cancer cells through up-regulating expression of CCAAT enhancer binding protein gamma (CEBPG) and formin like 3 (FMNL3) through miR-22 and miR-150-3p (Luo and Gui, 2020).

## Conclusion and Future Perspectives

Ovarian cancer is a comprehensive disease, but the pathogenesis has not been completely elucidated. Although substantial progress has been made in the diagnosis and treatment of ovarian cancer, unfortunately, the prognosis remains unsatisfactory. A growing number of ncRNAs have been identified to be involved in chemoresistance of ovarian cancer. Targeting ncRNAs, in combination with traditional chemotherapy or targeted therapy, may be a promising choice to combat drug resistance in advanced ovarian cancers. NcRNAs affect cell drug resistance through multiple mechanisms. In ovarian cancer, we reviewed EMT, drug efflux transporters, autophagy, cell cycle dysregulation, and DNA repair abnormality. At present, it has also received widespread attention that ncRNAs mediate exosomes to cause cell drug resistance.

A variety of methods are used to identify ncRNA that affect drug resistance, and the more commonly used methods include high-throughput analysis, silicon analysis, integrated analysis, bioinformatics, and expression arrays (Hartmaier et al., 2017; Cen et al., 2021). These technologies enable researchers to target the direction of tumor research, explore the mechanism of tumor occurrence and development, and explore the mechanism of clinical drug resistance. However, it is still a great challenge to select the critical target ncRNAs from the large number of candidates and there is still a long way to go for ncRNA to be used as clinical drug targets. Further translational studies or clinical trials are indispensable to develop ncRNAs-based therapeutics, which may ultimately provide potential approaches for overcoming ovarian cancer drug resistance.

## Author Contributions

XZ and SX designed the study. HL analyzed and interpreted the data, and wrote the original draft. JYu wrote this manuscript. DZ, CL, XG, and JYo edited and revised the manuscript. All authors have seen and approved the final version of the manuscript.

## Conflict of Interest

The authors declare that the research was conducted in the absence of any commercial or financial relationships that could be construed as a potential conflict of interest.

## Publisher’s Note

All claims expressed in this article are solely those of the authors and do not necessarily represent those of their affiliated organizations, or those of the publisher, the editors and the reviewers. Any product that may be evaluated in this article, or claim that may be made by its manufacturer, is not guaranteed or endorsed by the publisher.

## Abbreviations

PTX: paclitaxel
ADR: adriamycin
PARPi: poly-ADP ribose polymerase inhibitor
PFS: progression-free survival
ncRNAs: non-coding RNAs
miRNAs: microRNAs
lncRNAs: long non-coding RNAs
circRNAs: circular RNAs
MDR: multidrug resistance
EMT: epithelial-mesenchymal transition
piRNAs: PIWI-interacting RNAs
snoRNAs: small nucleolar RNAs
snRNAs: small nuclear RNAs
3′ -UTR: 3′ -untranslated region
mRNAs: messenger RNAs
DDP: cisplatin
PRKC: protein kinase C
PTEN: phosphatase and tensin homolog
PPP1R12A: protein phosphatase 1 regulatory subunit 12A
MST: STE20-like kinase
SAV1: protein salvador homolog 1
SFRP1: secreted frizzled-related protein 1
KCNMA1: potassium calcium-activated channel subfamily M alpha 1
FOXO3: forkhead box O3
TRIM31: tripartite motif containing 31
EZH2: zeste homolog 2
PTPN3: protein tyrosine phosphatase non-receptor type 3
ITGB8: integrin subunit beta 8
DDR1: discoidin domain receptor 1
NOTCH1: notch receptor 1
HMGA1: high mobility group AT-hook 1
RAD51: RAD51 recombinase
DNMT: DNA methyltransferase
AXL: AXL Receptor Tyrosine Kinase
APAF1: apoptotic peptidase activating factor 1
CIC: capicua transcriptional repressor
ING5: inhibitor of growth family member 5
XIAP: X-linked inhibitor of apoptosis
STAT3: signal transducer and activator of transcription
TRIM27: tripartite motif containing 27
SIK2: salt inducible kinase 2
DSB: double-strand breaks
RNASEH2A: ribonuclease H2 subunit A
FEN1: flap structure-specific endonuclease 1
SSRP1: structure specific recognition protein 1
SSA: single-strand annealing
HGSOCs: high-grade serous ovarian carcinomas
NHEJ: non-homologous end joining
NRP1: Neuropilin 1
RNAP II: RNA Polymerase II
ceRNA: competitive endogenous RNA
UCA1: urothelial cancer associated 1
ABC: ATP binding cassette
HOTAIR: HOX antisense intergenic RNA
ATG7: autophagy related 7
VPA: valproic acid
CCAT1: colon cancer associated transcript 1
MALAT1: metastasis-associated lung adenocarcinoma transcript 1
ZEB1: zinc finger E-box binding homeobox 1
Gal-1: galectin 1
FOXR2: forkhead box R2
SCAI: suppressor of cancer cell invasion.
